# Indoor Pedestrian Navigation Based on Conditional Random Field Algorithm

**DOI:** 10.3390/mi8110320

**Published:** 2017-10-30

**Authors:** Mingrong Ren, Hongyu Guo, Jingjing Shi, Juan Meng

**Affiliations:** 1 College of Automation, Faculty of Information Technology, Beijing University of Technology, Beijing 100124, China; 2009zsf@emails.bjut.edu.cn (H.G.); shijingjing123@emails.bjut.edu.cn (J.S.); S201761125@emails.bjut.edu.cn (J.M.); 2 Engineering Research Center of Digital Community, Ministry of Education, Beijing 100124, China; 3 Beijing Key Laboratory of Computational Intelligence and Intelligent Systems, Beijing 100124, China

**Keywords:** indoor localization, pedestrian navigation, map matching, inertial sensors, conditional random fields

## Abstract

Foot-mounted micro-electromechanical systems (MEMS) inertial sensors based on pedestrian navigation can be used for indoor localization. We previously developed a novel zero-velocity detection algorithm based on the variation in speed over a gait cycle, which can be used to correct positional errors. However, the accumulation of heading errors cannot be corrected and thus, the system suffers from considerable drift over time. In this paper, we propose a map-matching technique based on conditional random fields (CRFs). Observations are chosen as positions from the inertial navigation system (INS), with the length between two consecutive observations being the same. This is different from elsewhere in the literature where observations are chosen based on step length. Thus, only four states are used for each observation and only one feature function is employed based on the heading of the two positions. All these techniques can reduce the complexity of the algorithm. Finally, a feedback structure is employed in a sliding window to increase the accuracy of the algorithm. Experiments were conducted in two sites with a total of over 450 m in travelled distance and the results show that the algorithm can efficiently improve the long-term accuracy.

## 1. Introduction

Determining the position of a person is required in many applications, such as first responders, mine workers and indoor civilians. A global navigation satellite system (GNSS) is essential for outdoor navigation. However, GNSS signals are unavailable indoors for attenuation and creation of multiple paths. Many technologies, including ultrawideband (UWB) [1], WiFi [2], radio frequency identification (RFID) [3], inertial navigation [4,5,6], etc., have been studied for indoor navigation. Unlike outdoor navigation, no single approach has been shown to be most suitable for the majority of applications [7]. UWB, WiFi and RFID are infrastructure-dependent technology and in non-line-of-sight conditions, they suffer great errors in localization. Inertial navigation systems (INS) are self-contained, because they do not receive or transmit any signals. Low-cost micro-electromechanical systems (MEMS) sensors suit the requirement for consumer applications with a low cost, such as pedestrian navigation [8]. However, the errors of low-cost MEMS-based pedestrian navigation systems (PNS) will accumulate due to bias and noise in measurement. Although the zero-velocity update (ZUPT) method is intended to restrict positional errors, this technology cannot correct the azimuth error. Due to the inability to observe the yaw error, the heading drift becomes the main factor that causes the estimated positional error. 

In order to reduce yaw error, several methods are used. Map matching proposed by the authors of [9,10,11,12] show great improvement in estimating positions. In a study [9], Cardinal Heading Aided for Inertial Navigation System (CHAIN) is employed to mitigate heading drift. The algorithm uses the simple notion that users are likely to walk in one of four cardinal headings. In another study [10], a confidence of CHAIN is utilized to update the weight of particles. The approach reduces the number of particles and improves the performance of the system. In another study [11], this update in weight is assigned by the discrete dominant direction (DDD) rule and human behavior. Eight directions are employed according to the Gaussian distribution. In [12], only the building outline is needed for filtering particles to improve the accuracy. Particle filters need to compute the posterior density $p(s_{t-1}|z_{1},\cdots ,z_{t-1})$ by a set of weighted particles, where $s_{t-1}$ is the real state (e.g., the location of a person), while $z_{1},z_{2},\cdots$ and $z_{t-1}$ are the observation variables. Checking the compliance of every particle against the map constraints increases the computational cost of this algorithm.

The conditional random fields (CRFs) were applied to pedestrian indoor navigation by Xiao et al. [13] and Bataineh [14]. Both of them have good performance. The algorithm has two advantages. Firstly, the computation is efficient. Secondly, it can capture arbitrary constraints between observations. In a study [13], the map is divided into identical squares with edge lengths of e, which is set as the narrowest corridor in most buildings (e.g., 0.8 m). Two feature functions were used. One describes the relationship of the orientation and length of two observations in two consecutive states. Another involves the rotation of observation $z_{i}$ by a pre-defined degree. The third feature function is optional, which takes the received signal strength (RSS) fingerprint data into account. The fourth feature function can also be added to the model if the building is equipped with cameras or other sensors. Extensive experiments show that this method outperforms state-of-the-art approaches, such as hidden Markov model (HMM) and particle filters. In [14], the length of cell size was set as 1 m through the experiments, while a buffer size of 2 cells was used for transitions to the neighboring cells. Only one feature function is used, which is based on the distance between the observed and the candidate states. However, another technique called improved heuristic drift elimination (iHDE) [15] is also used to reduce heading errors. The experiments show that the CRF + iHDE algorithm is superior to the sole iHDE algorithm. In [13], a distance of 200 m was tested for different types of cell phones, while 352 m was tested on wrist-worn inertial measurement units in another study [14]. In [14], only 25 states were chosen for every observation, while in another study [13], the vertices in the whole indoor map are chosen as states for one observation. Although the former is more computationally effective, it can only correct distance errors less than 3 m. The latter can correct errors as large as the whole indoor distance in theory.

The map-matching technique based on conditional random fields described in this paper aims to decrease the number of states for every observation and to improve the accuracy of the system. The pre-defined states are squares with edge lengths of e = 0.8. Only four states are chosen for each observation. The observations are chosen as positions from INS. The next position is obtained from fixed length intervals (i.e., 0.8 m), instead of being based on step length or same time intervals. 

## 2. System Description

**Figure 1:**
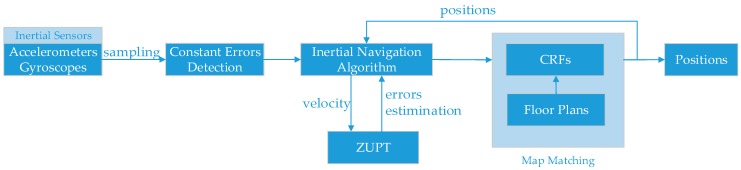
System architecture.

The system architecture is described in Figure 1. Inertial sensors, including three accelerometers and three gyroscopes, are used to determine locations. As magnetometers are susceptible to disturbance, we do not use them to obtain the system heading. Generally speaking, the whole system consists of 5 sections. The first part involves the data sampling from inertial sensors, which is conducted every 0.01 s by computers. The second part involves the constant errors detection algorithm, with the gyroscope constant errors detected in this stage. The third part is inertial navigation algorithm, which is based on traditional inertial navigation equation [16]. The fourth part is the ZUPT algorithm, which has already been detailed in [16]. The fifth part is the CRFs-based map matching method. There is feedback between CRFs and inertial navigation algorithm.

### 2.1. Detection of Constant Errors

As constant errors of gyroscopes have an enormous effect on the accuracy of the system, these errors cannot be estimated by the Kalman filter used in the ZUPT stage, which was described in detail in study [16]. Therefore, some methods must be performed to deal with this problem. We propose a simple but very useful technique to estimate the errors. When the system is strapped onto a user’s shoe, it is very convenient for the user to stand still for a while. In our experiments, we require the minimum time intervals are 6 s. During these time intervals, if the standard deviation of the gyroscopes is lower than a threshold value, the mean values of the outputs of the gyroscopes can be roughly recognized as constant errors. When the user begins to move around, the constant errors should be subtracted from the outputs of the gyroscopes. We call this process “the constant errors detection”. The constant errors that are not estimated will cause heading errors and will eventually affect the positional errors, which will be corrected by the proposed CRFs algorithm. 

### 2.2. Inertial Navigation Algorithm

To calculate a pedestrian’s position, three basic navigation equations are used. Those are velocity, attitude and position differential equations.

(1) $${\dot{V}}^{n}=C_{b}^{n}f_{ib}^{b}+g$$

(2) $${\dot{C}}_{b}^{n}=C_{b}^{n}(\omega_{nb}^{b}\times )$$

(3) $$R^{n}(k)=R^{n}(k-1)+V^{n}(k)T_{s}$$

where $C_{b}^{n}$ is the transformation matrix from the *b* frame to the *n* frame and $f_{ib}^{b}$ is the specific force in the body frame. The body frame is defined as a right-handed (x,y,z) Cartesian coordinate system (x means forward, y means left, z means upward). The navigation frame is defined as east-north-up (ENU). We use the subscripts b (body) and n (navigation) to denote the project of a vector in a corresponding frame. $\omega_{nb}^{b}$ represents the angular rate of the body frame relative to the navigation frame. $\omega_{nb}^{b}\times$ represents the cross product matrix of $\omega_{nb}^{b}$. $T_{s}$ is the sampling period.

### 2.3. ZUPT

When a person is walking, his/her foot will usually stay on the ground for approximately 0.1–0.3 s. We call these gaps the zero-velocity intervals. If the errors can be compensated during those periods, the accuracy of the foot-mounted pedestrian system can be improved greatly. In study [16], we detailed how to detect these intervals and verified the sensor bias errors, attitude errors and positional errors. Speed of a pedestrian is used to detect the zero velocity intervals, also the hidden Markov model was employed to identify acceleration intervals, deceleration intervals and zero velocity intervals. This method can perform well in various motion models. The performance of the system can be highly improved by these techniques. However, the heading error cannot be observed and cannot be estimated, which makes it the main error that affects the accuracy of the system. Therefore, we propose the CRFs-based map-matching method. 

### 2.4. Linear-Chain CRFs 

CRFs [17] are undirected probabilistic models used to compute the probability p(s|z) of a possible output $s=(s_{0},s_{1},\cdots s_{n})$ when given the input $z=(z_{0},z_{1},\cdots z_{n})$. A special form of CRF is known as a linear chain structure. In that case, the output variables are modeled as a sequence. Therefore, the conditional probability p(s|z) can be written as:

(4) $$p(s|z)=\frac{1}{Z(z)}\prod_{j=1}^{n}\Psi_{j}(s,z)$$

where n is the number of factors. We noted that there are n+1 observation variables, because $s_{j-1}$ and $s_{j}$ are connected in one factor. Each factor $\psi_{j}\geq 0$ relates to a potential function that incorporates different features $f_{i}$ of the required part of the input and output.

(5) $$\Psi_{j}(s,z)=\exp(\sum_{i=1}^{K}\lambda_{i}f_{i}(s_{j-1},s_{j},z,j))$$

p(s|z) can also be written as:

(6) $$p(s|z)=\frac{1}{Z(z)}\exp(\sum_{j=1}^{n}\sum_{i=1}^{K}\lambda_{i}f_{i}(s_{j-1},s_{j},z,j))$$

Z(z) is called the normalization factor, which is calculated as follows:

(7) $$Z(z)=\sum_{z^{\prime }}\prod_{j=1}^{n}\psi_{j}(s,z^{\prime })$$

The summarization is conducted for all possible output sequences and the normalization factor ensures that p(s|z) is a probability measure. Equations (6) and (7) form a linear-chain CRF. Given the input vectors z and the potential function $\Psi_{j}(s,z)$, we need to find the most likely hidden states $s^{*}$ as follows:

(8) $$\begin{array}{ccc} s^{*} & = & \arg\underset{s}{\max}\;p(s|z)\\ & = & \arg\underset{s}{\max}\prod_{j=1}^{n}\Psi_{j}(s,z)\\ & = & \arg\underset{s}{\max}(\sum_{j=1}^{n}\sum_{i=1}^{K}\lambda_{i}f_{i}(s_{j-1},s_{j},z,j)) \end{array}$$

In Equation (8), we only need to compute the non-normalized probability. This can greatly reduce the computational complexity. The Viterbi algorithm [18] provides a good way to compute the most probable values in Equation (8). 

The steps of Viterbi algorithm can be simply summarized as follows:

(1) Initialization: Compute the non-normalized probability of the first position for all states, where m is the number of states.
  (9) $$\delta_{1}(j)=\sum_{i=1}^{K}\lambda_{i}f_{i}(s_{0}=start,s_{1}=j,z),j=1,2\cdots m$$
(2) Recursion: Compute and find the biggest the non-normalization probability to the position p=2,3,⋯n over all states l=1,2,⋯,m, at the same time record the state label $\Psi_{p}(l)$ that has the largest value.
  (10) $$\delta_{p}(l)=\underset{1\leq j\leq m}{\max}\{\delta_{p-1}(j)+\sum_{i=1}^{K}\lambda_{i}f_{i}(s_{p-1}=j,s_{p}=l,z,)\},l=1,2\cdots m$$
  (11) $$\Psi_{p}(l)=\arg\underset{1\leq j\leq m}{\max}\{\delta_{p-1}(j)+\sum_{i=1}^{K}\lambda_{i}f_{i}(s_{p-1}=j,s_{p}=l,z,)\},l=1,2\cdots m$$
(3) When p=n, find the largest $\delta_{n}(j)$ and record the terminal track state label $s_{n}^{*}$.
  (12) $$\underset{s}{\max}\;p(s|z)=\underset{1\leq j\leq m}{\max}\delta_{n}(j)$$
  (13) $$s_{n}^{*}=\arg\underset{1\leq j\leq m}{\max}\delta_{n}(j)$$
(4) Calculate the final output sequence as follows:
  (14) $$s_{p}^{*}=\Psi_{p+1}(s_{p+1}^{*}),p=n-1,n-2,\cdots ,1$$

The complexity of the algorithm is $O(n\times {\left|m\right|}^{2})$.

### 2.5. Map Pre-Processing

To use CRFs, we need to define states and feature functions for every observation. However, indoor maps should be processed to get discrete states before this. The obtained indoor maps are in an image format, which is not suitable for the map-matching algorithm. First, we use a software called MapInfo to convert the image into a digital format. After this, the map is divided by a set of square cells, with the vertices being possible states. We get the vertices using the following equations:

(15) $$\left\{ \begin{array}{l} map\_x=map\_x0+0.8k\\ map\_y=map\_y0+0.8k \end{array}\right.,k\in (0,1,2\cdots )$$

where map_x0 and map_y0 are given as initial positions in the digital map. 

**Figure 2:**
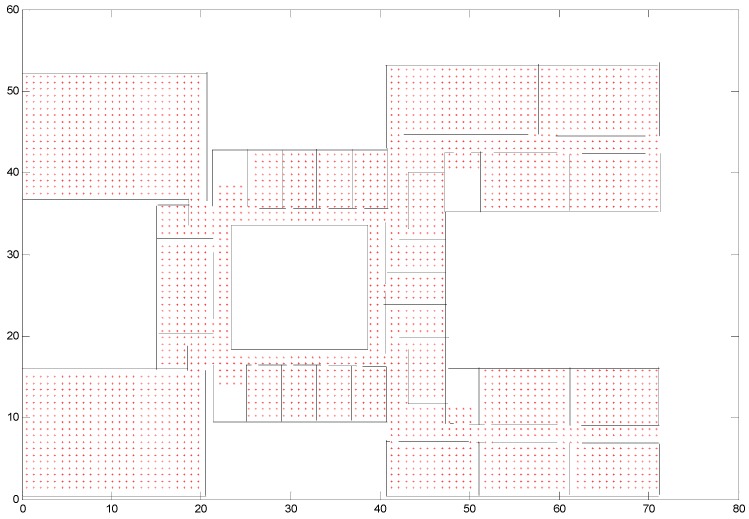
States in Economics and Management Building.

If the vertices are too close to the wall, they are no longer considered as states. In this paper, we set the threshold value as 0.2 × *a* (*a* is the square length). If the distance between a vertex and the wall is smaller than 0.2 × *a*, the vertex is removed. We should bear in mind that there are some restrictions between states. For example, if the edge between two vertices crosses a wall, the states cannot transition from one vertex to another. A higher accuracy will be obtained if more time is spent on the map processing stage. Figure 2 shows states in the Economics and Management Building in Beijing University of Technology, with the edge length of a square cell being 0.8 m. 

### 2.6. Definition of States and Choice of Observations 

CRF models use a set of input observations, such as positions and headings, to predict hidden variables. The predicted trajectories are located exactly on one of the states. In this step, we should determine what observations can be used in CRFs and how to choose hidden states for each observation. 

The hidden states for observations based on step length should consider that step length is not same for different individuals and even a same person will have different step lengths at different walking speeds. In [13], a buffer size is chosen for every observation. However, knowing that ZUPT has already estimated the accelerometer errors, the length from one position to another position is quite accurate. The errors left uncorrected are gyroscope drifts and azimuth errors. We can use this characteristic to further reduce the number of hidden states for every observation. 

The observations are chosen as positions that are a fixed length from each other. The equation is shown as follows:

(16) $$d=\sqrt{{\left(z_{x\_pi}-z_{x\_pj}\right)}^{2}+{\left(z_{y\_pi}-z_{y\_pj}\right)}^{2}},$$

where pi and pj are two positions; $z_{x\_pi}$ represents the x coordinate in position pi; $z_{y\_pi}$ represents the y coordinate in position pi; $z_{x\_pj}$ represents the x coordinate in pj position; and $z_{y\_pj}$ represents the y coordinate in position pj. Supposing that pi is the last observation, if the distance between the position pi and pj is equal to d, pj is chosen as the next observation. d is equal to the edge length between two consecutive states. 

We must emphasize that the observations are not chosen as step lengths. d differs from step length the majority of the time. 

When the observations are determined above, hidden states are simply chosen as the nearest 4 vertices around every observation. 

### 2.7. Definition of Feature Functions

A feature function defines the extent to which the observation supports two consecutive states. A high value of the feature function indicates stronger support [13]. As the length of the positions is used to choose observations, the feature function is selected as the orientation of two consecutive observations:

(17) $$f(s_{t-1},s_{t},z_{t}^{\theta })=\ln\frac{1}{\sigma_{\theta }\sqrt{2\pi }}-\frac{{\left(z_{t}^{\theta }-\theta (s_{t-1},s_{t})\right)}^{2}}{2\sigma_{\theta }^{2}}$$

where $\sigma_{\theta }$ is the orientation variance of two consecutive observations and we set $\sigma_{\theta }$ = 22.5°in our INS system. $\theta (s_{t-1},s_{t})$ is the orientation of the edge between states $s_{t-1}$ and $s_{t}$, while $z_{t}^{\theta }$ is the orientation of two consecutive observations. This is the only feature function used in CRFs. 

Therefore, Equation (8) can be rewritten as:

(18) $$s^{*}=\arg\underset{s}{\max}(\sum_{j=1}^{n}f(s_{j-1},s_{j},z_{j}^{\theta },j))$$

A sliding window is used to select the most probable states and the length of the window is set as 2.4 m. Therefore, four positions (n=4) need to be estimated using CRF algorithm in every window, with the window sliding 0.8 m. 

### 2.8. Feedback Technique

Another technique employed in this paper is the feedback of the output sequence of CRFs to the inertial navigation algorithm to obtain more accurate positions as shown in Figure 1. The output sequence of CRFs is the estimated positions, denoted as $s_{x}$ and $s_{y}$, which feedback to inertial navigation algorithm to replace the raw positions from inertial navigation algorithm, and the subsequent positions are recalculated from these estimated positions:

(19) $$R_{x}^{n}(k)=s_{x}$$

(20) $$R_{y}^{n}(k)=s_{y}$$

(21) $$R^{n}(k+1)=R^{n}(k)+V^{n}(k)T_{s}.$$

where $R_{x}^{n}(k)$ means x coordinate in position $R^{n}(k)$. $R_{y}^{n}(k)$ means y coordinate in position $R^{n}(k)$. Experiments in Section 3 will show the effect.

## 3. Experiments

**Figure 3:**
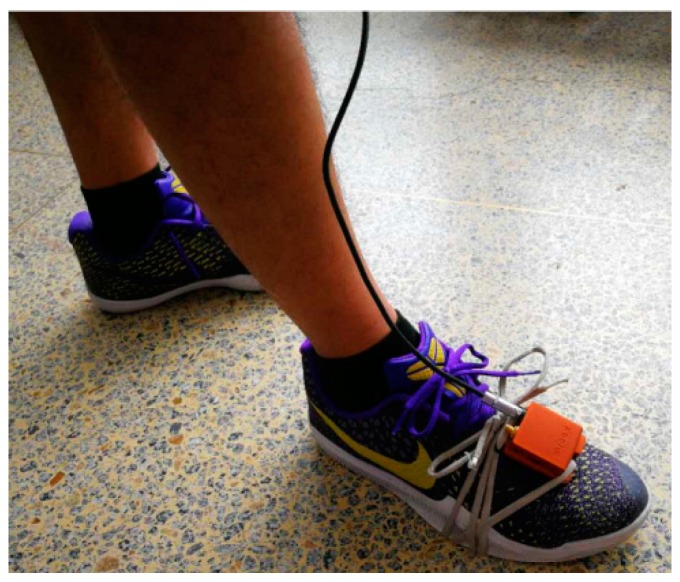
Inertial measurement unit (IMU) strapped on to a shoe.

The inertial measurement unit used in the experiments is the model MTI-G from Xsens Technologies, which is the same as [16]. The inertial measurement unit (IMU) is strapped on to a shoe, as shown in Figure 3.

### 3.1. Robustness for Different Individuals and Different Indoor Environments

**Figure 4:**
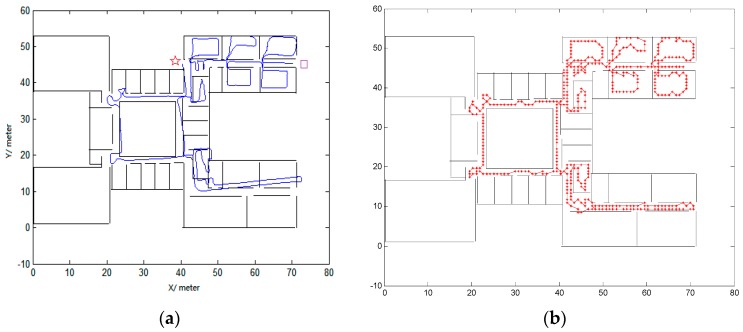
Comparison of trajectory with (**a**) being the raw trajectory and (**b**) being the trajectory using conditional random fields (CRFs).

First, the experiments were tested in the second floor of the Economics and Management Building in Beijing University of Technology. The average width of the corridors in this building is 2 m. The first trajectory was conducted by a male with a height of 1.7 m. The travelled distance is approximately 423 m. Figure 4 shows the comparison of trajectories. The left figure is the raw trajectory. The purple square is the start point and the red star is the end point. From the left figure, we can see that the trajectory is accurate at the beginning of the track, but the errors begin to increase with time and some trajectories even cross the walls. The right figure shows the trajectory using the proposed algorithm in this paper. We can easily identify whether the pedestrian is in the room or in the corridor. There are no wall crossings during the track.

**Figure 5:**
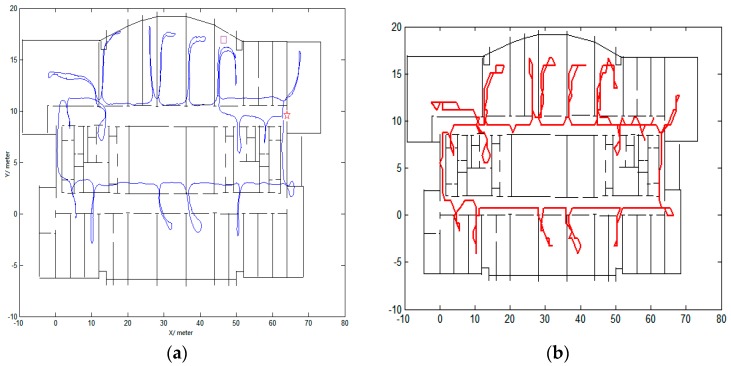
Comparison of trajectory carried out in another site by another person with (**a**) being the raw trajectory and (**b**) being the trajectory using CRFs.

The second trajectory was conducted on the 10th floor of Science Building in Beijing University of Technology by a female with a height of 1.6 m. The area of the building is approximately 80 × 23 m^2^. The width of the narrowest corridor is 0.8 m, the width of the widest corridor is 3 m, while the width of the other corridors is 2.5 m. The travelled distance is approximately 470 m. Figure 5 shows the comparison of trajectories. The start point and end point are defined as being same as in Figure 4. The left figure is the raw trajectory, from which we cannot correctly locate the pedestrian and the errors are large. The right side of Figure 5 gives the trajectory calculated using the algorithm proposed in this paper. The accuracy has improved greatly.

In study [13], the experiments are carried out on a smart phone using only the accelerometer and magnetometer measurements. The average tested length is about 200 m and overall the error is around 4 m in the 97th percentile. In [14], the inertial measurement requires a wrist-worn unit composed of three accelerometers and three gyroscopes. The average tested length is about 350 m, but the experiments are only conducted along the corridors and no room entry events. Therefore, there are no obstacles crossed and the error is about 2 m for the 90th percentile. 

Our inertial measurement unit is strapped on a pedestrian’s foot, with three accelerometers and three gyroscopes used to determine the position. The average tested length is about 450 m. The tracks are very winding as can be seen from Figure 4 and Figure 5. We do not use labels placed along corridors and within rooms to evaluate the absolute errors as conducted in [13]. We only evaluate the percentage of matched trajectories entering the correct room [13]. We have also conducted several experiments for different pedestrians and in different indoor environments, obtaining an overall accuracy of 98%. There are four reasons for this high accuracy. First, it is related to the pre-processing of the map. For example, states located near the walls are deleted and states that cross walls are not allowed. Secondly, the effectiveness of ZUPT and constant errors detection contribute greatly, in which reduces the divergence of the raw trajectory as previously seen in [13]. Thirdly, a feedback structure is used to obtain the high accuracy. Fourthly, fixed length rather than step length is employed for the two successive observations.

### 3.2. Comparison

Another experiment is conducted on the 10th floor of Science Building by a male with a height of 1.8 m. We will first compare our algorithm with non-feedback structure.

**Figure 6:**
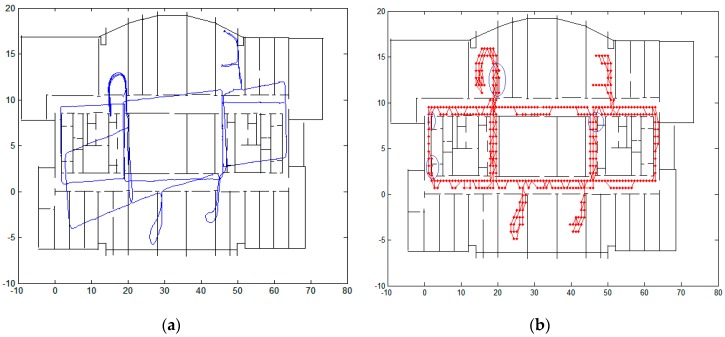

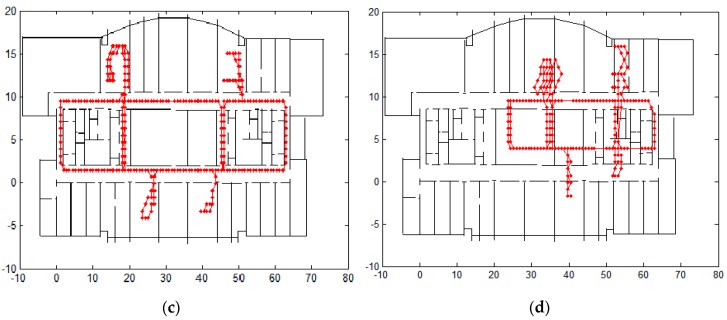
Comparison of trajectory: (**a**) raw trajectory; (**b**) trajectory using CRFs without feedback; (**c**) trajectory using proposed method; and (**d**) trajectory using step length as observations.

A closed-loop structure is necessary for the algorithm to obtain high accuracy. Figure 6 gives the comparison of the raw trajectory, trajectory using CRFs without closed-loop structure and trajectory using the proposed algorithm in this paper. The blue circles in Figure 6b show some mismatched trajectories, which have crossed the walls. The overall rate of mismatch is 10%. When positions from CRFs are fed back to the inertial navigation algorithm, the accuracy of the algorithm improves greatly and the mismatched is reduced to 2%. 

We also use this experiment to compare the results when the length between two successive observations is chosen as the step length and the number of the states for each observation is still chosen as 4 states. The trajectory is shown in Figure 6d. The average step length of the pedestrian is about 1.2 m, which is bigger than the edge length of the square cell (0.8 m). Therefore, the calculated trajectory deviated greatly from the true trajectory. The advantage of the proposed algorithm is that we do not need to change the length of the square cell to adjust to different individuals. Furthermore, only 4 states rather than 25 states in [14] are chosen for each observation. 

## 4. Conclusions

We demonstrated a new algorithm using conditional random fields. The algorithm can be applied in various sites and used by different individuals. The complexity of the algorithm is lower when compared with particle filters or other existing CRFs algorithms. The success of the algorithm is partly based on ZUPT and constant errors detection, which enable the estimated length of trajectories to be relatively accurate. The main contribution of the algorithm is that the length between two successive observations is equal to the length of two successive states, so only 4 states for each observation are required to obtain high accuracy.
